# Medication management for people with dementia in primary care: description of implementation in the DelpHi study

**DOI:** 10.1186/1471-2318-13-121

**Published:** 2013-11-13

**Authors:** Thomas Fiß, Jochen René Thyrian, Diana Wucherer, Grit Aßmann, Ingo Kilimann, Stefan J Teipel, Wolfgang Hoffmann

**Affiliations:** 1 German Center for Neurodegenerative Diseases (DZNE), Rostock/Greifswald, Germany; 2 Institute for Community Medicine, University of Greifswald, Ellernholzstr 1-2, 17489 Greifswald, Germany; 3 Department of Psychosomatic Medicine, University Hospital Rostock, Gehlsheimer Straße 28, 18147 Rostock, Germany

**Keywords:** Home medication review, Drug related problems, Dementia, Potentially inappropriate medication, Health services research

## Abstract

**Background:**

As the population ages, the relative and absolute number of age-associated diseases such as dementia will increase. Evaluation of the suitability and intake of medication and pharmacological treatment is an important aspect of care for people with dementia, especially if they live at home. Regular medication reviews and systematic cooperation between physicians and pharmacists are not common in routine care. Medication management (MM), based on such a comprehensive home medication review could help to reduce drug-related problems and costs. The present article presents a medication management specifically for the application in the ambulatory setting and describes its implementation as part of a larger trial.

**Methods/design:**

A home medication review (HMR) and MM is implemented as part of the DelpHi study, a population based prospective, cluster-randomized controlled intervention study to test the efficacy and efficiency of the implementation of a collaborative care model in primary care.

Participants: people with dementia (PWD) and their caregivers are recruited by the patient’s general practitioner. Inclusion criteria are a positive screening result for dementia, living at home and regular intake of drugs. PWD are asked to specify their regular pharmacy which is asked to participate in the study, too.

Intervention: a comprehensive HMR is conducted as computer-assisted personal interview by specifically qualified Dementia Care Manager (DCM) at the people’s home. It includes detailed information about drugs taken, their storage, administration, adherence and adverse events. The MM is conducted in cooperation between DCM, pharmacist and general practitioner and consists of a pharmaceutical evaluation, pharmaceutical recommendations and their application. Pharmacists are trained and provided with regularly updated information. The MM is designed to give information and recommendations concerning antidementia drugs, occurrence of drug related problems, intake of anticholinergic drugs, potentially clinically relevant drug-drug-interactions, adverse drug events and medication adherence.

**Discussion:**

The DelpHi-approach for medication management employs comprehensive instruments and procedures in the primary care setting under routine care conditions, and this approach should be useful in improving pharmacotherapy as part of the comprehensive treatment and care for people with dementia.

**Trial registration:**

The trial is registered at ClinicalTrials.gov, number NCT01401582.

## Background

Western health care systems struggle with the impact of demographic changes. The aging of the population will lead to an increase in both the relative and the absolute number of age-associated diseases, such as dementia. The term “dementia” refers to a clinical syndrome that is characterized by the loss of cognitive functions, such as delayed recall, working memory, orientation, language or executive functions, and impaired activities of daily living (ICD-10). The number of people with dementia (PWD) in Mecklenburg Western Pomerania, a federal state in the north-east of Germany, is expected to increase by 91.1% between 2005 and 2020 [1]. Actual dementia-centered care is insufficient. Recently, Thyrian et al. identified several important working fields to improve the treatment and primary care for people with dementia in Germany [2]. For example, there is a need for adequate diagnosis and treatment. There are several guidelines, such as the German S3-guidelines and the British NICE diagnosis guideline for dementia, which recommend a multistep algorithm for dementia diagnosis [3]. The algorithm includes the assessment of a comprehensive clinical history, neurological and psychiatric examination, basic laboratory and structural neuroimaging An important part of the assessment of the clinical history is a review of the medical history, and as such, this is an obligatory element in the algorithm for dementia diagnosis. However, the aspect of medication review is limited to negative side effects of anticholinergic drugs on cognition [4,5]. Anticholinergics compromise cognition and may lead to a misinterpretation of clinical assessments. A systematic procedure for medication review and detection of drug-related problems is lacking. PWD may not be a reliable information source for medication review because cognitive impairment per se is associated with a reduced ability to manage pharmacotherapy [6].

Drug choice and regular evaluation as a part of medication management (MM) plays an important role in care-giving for PWD. In general, the intake of several drugs is associated with the risk of drug-related problems (DRP) as well as a high cost for the treatment of adverse events [7,8]. DRPs comprise several categories, including drug-drug interactions (DDIs), adherence problems, inappropriate drug choice and inappropriate usage by the patient [9]. The DRP-category “inappropriate medication” is of specific relevance for the PWD. Anticholinerg drugs [4] often affect patients’ cognition adversely, increase treatment costs due to increased hospitalizations, and may cause falls [10-13]. Polypharmacy is a general co-factor for every DRP category and can be a major co-factor for increased, non-disease specific cognitive decline and reduced abilities in the activities of daily living in elderly patients with dementia. Sedative drugs such as benzodiazepines are strongly associated with falls and hip fractures [14]. Even with the appropriate prescription of every medication, every additionally administered drug increases the risk of clinically relevant drug-drug interactions [15]. DDIs can result in drug-induced hospitalizations, elevated mortality, and decreased quality of life. Even DDIs not leading to hospitalizations often compromise patients’ adherence to their drug therapy [16]. Recently, Rottenkolber et al. estimated total costs of 400 Mio. € p.a. for drug-associated hospitalizations in Germany [7]. Another problem in geriatric pharmacotherapy is frequent off-label prescribing [17]. This activity leads to the legal dilemma between the necessity of therapy and the limited therapeutic options, as many medications are not approved for use in geriatric patients, resulting in increases in the occurrence of off-label prescription.

A comprehensive medication review could help to improve the care for PWD. There is evidence for positive effects of such a review on medication-related and clinical outcomes, such as hospitalizations and mortality, in elderly people [18-20]. However, neither the German S3-guidelines nor the NICE-guidelines specify procedures for a medication review or quality management as part of the diagnosis or treatment of dementia.

The first part of a medication review is a general interview about the patient’s drug intake behavior and a complete assessment of all drugs taken by the patient, including the intake modalities (over-the-counter (OTC) and prescribed) [21]. The patient’s medication list can be used during this step, however this information is not always reliable and only 70% of elderly provide a patient medication list. For those who do have a list, OTC drugs are often not documented [22]. OTC medications are important in drug management, as they often cause clinically relevant drug-drug interactions or further DRPs.

Maidment et al. concluded that “medication management in dementia is a broad concept that should encompass a complete review of medication, including assessment of indication, dosage, interactions and continued need” [23]. Medication management may help to improve quality of care. Earlier publications of the American Health Association classified medication management as one of the most important aspects of interventions in disease management [24]. However, detailed procedures for medication management in dementia are still not specified. Medication management is difficult since there are interface problems within the health care system. For example, in Germany, medication is prescribed by physicians, sold by pharmacists and purchased by PWD and their caregivers. Also, a nursing service might coordinate drug administration. The German health care system does not provide a systematic exchange of information between these parties, so that counseling provided to the patient or caregiver by one party is limited. For example, the prescription given to the pharmacist contains neither an indication for drug intake nor a prescribed dosage [25], the physician does not necessarily know what OTC drugs are sold or taken. There are no digital interfaces, such as electronic health records. Collaborative health care networks, medication management and IT-support with electronic health records can reduce incomplete information on the side of the General Practitioner (GP) and other physician(s) involved in the treatment of an elderly patient. This counts even more for patients suffering from a dementia syndrome [23,26].

Presently, there is a lack of instruments available for medication review and for medication management even though the SOAP (Subjective and Objective problem Assessment, and Planning of improvement) scheme, which is based on pharmaceutical care, can help with the medication management process [22]. However, medication management is not a well-implemented modality in clinical and primary dementia care in Germany. Therefore, the aims of this paper are:

1. To develop procedures and instruments for a home medication review (HMR) and medication management process for PWD living at home, specifically for Germany.

2. To describe the implementation of such an HMR and medication management process as one module in a primary care intervention trial designed to deliver optimum care to PWD living at home.

## Methods/design

HMR with a subsequent MM is part of the complex intervention in the DelpHi study (dementia: life and person-centred support in Mecklenburg Western Pomerania) [27]. The DelpHi-study is a population based prospective, cluster-randomized controlled intervention study. Details of the study design are described in detail elsewhere [27]. In the course of this study, the MM described in this article will be tested for its efficacy by comparing an intervention group that receives MM versus a control group that does not receive MM. The complex intervention also comprises caregiver counselling and providing a subsidiary support system for the care of the PWD, which is not part of this article.

### Participants

The DelpHi-study recruits participants with suspicion of dementia (DemTect <9 [28]) in GP practices. People 70 years or older, living at home, having sufficient knowledge of german and having a regular intake of drugs are included in the HMR and MM. Participants are asked to name a caregiver who is then invited to participate in the trial as well as to designate their regularly visited pharmacy.

The participating pharmacies are qualified to conduct the MM in a personal training course and are given comprehensive information on the study and further advice on the MM. The topics covered are:

(a) DelpHi study procedures and standard operating procedures (SOPs),

(b) basic principles of medication management,

(c) basic principles in the context of drug-related problems,

(d) clinical symptoms of dementia,

(e) special DRPs associated with dementia,

(f) the pharmacists’ role in the primary health care network,

(g) hands-on exercises with case-reports

Furthermore, the study pharmacist is available by telephone for clarification of further questions. The training was accredited by the chamber of pharmacists of Mecklenburg Western Pomerania.

If the pharmacy chosen by the participant does not take part in the MM part of DelpHi or the PWD does not name a pharmacy, the HMR of the respective participant will be conducted by the study team pharmacist. The participants in the control group receive care as usual (the pharmacist is not actively enrolled in the study).

### Data assessment

The baseline assessment for each PWD is conducted as an interview by specifically qualified Dementia Care Manager (DCM) [29] in the patient’s household. The aim of the interview is to capture the real medication care situation of people with dementia and the PWD is the primary interview partner. The informal caregiver (if present) can assist in answering the questions. In case of missing data other available proxies like participating GPs or a nursing service are used.

In addition to the HMR described in this article the following data are assessed: sociodemography; health care utilization; vital status using the STEP-screening [30]; (instrumental) activities of daily living; social integration; information on dementia (course and causes); quality of life; depression and cognitive status (SIDAM [31]).

Due to the cognitively demanding nature of the interviews, cognitive testing and HMR are conducted apart from the baseline assessment at a second home visit.

### Home medication review

The HMR is a computer-assisted personal interview (CAPI) delegated to specially qualified nurses [32]. To obtain full information about medication, a “bathroom cabinet review” is included. This comprises the look at all the medication available in the house, prescription drugs and OTC. If drugs are administered by a nursing service, the DCM contacts the nursing service and obtains a copy of the patient’s medication list. According to a previous review on the global dimensions of home medication in epidemiology and health care research [32] our HMR consists of data on:

● Drugs: brand name; pharmaceutical identifying number (a special number for the German market which allows identifying a given preparation and abstract additional information from pharmaceutical databanks); active substance, pharmaceutical form, if possible; daily dosage taken; day and time of drug intake, timing of drug intake relative to a meal; intake option (e.g., regular intake, emergency drug, or no longer needed); prescribing medical practitioner; medical indication for drug intake; the actual sales price.

● Drug storage: Where are the drugs stored (to ensure correct storage conditions, such as temperature and air humidity)? Additionally, the interviewer checks whether all of the drugs listed can be found in the named storage place as recorded.

● Drug administration: Who is available to assist with drug intake? Is a professional nursing service involved? Does the patient visit a preferred pharmacy and GP (to have a contact person if interfering drugs are identified)? Is drug administration supported by a relative or other informal caregiver? Does he or she need additional qualification? Is a medication list available, is it complete (e.g., over-the counter drugs; all regularly taken drugs as well as drugs taken on demand)? Is a drug dispenser used (as an adherence supporting strategy)?

● Drug adherence: multiple intakes due to forgetfulness are checked in addition to other adherence categories (e.g., complete discontinuation, drug holidays, and single skipped dosages). To measure this the Morrisky-Score, the MARS scale, and the CQR are available [33-35] and the German short form of the MARS scale for adherence measurement is used [36].

● Adverse drug events according to self-reports.

● Daily fluid intake and the type of drink consumed (to discover potential interactions: alcohol/grapefruit juice).

All information is entered in electronic case reporting forms and stored in a study database [37]. Upon completion of the HMR the system compiles a standardized interview report that includes the medication list. The DCM initiates a comprehensive medication management by printing a structured documentation which is forwarded to the patient’s pharmacy and the treating GP.

### Medication management/intervention

The Medication management is defined as a systematic, stepwise approach for the detection of DRPs and solution of DRPs, systematic drug documentation in close collaboration between DCM, pharmacist and GP.

Step 1 Pharmaceutical evaluation

After an initial analysis of the pharmacotherapy, the pharmacist prepares a list that addresses relevant drug-related problems. The pharmaceutical evaluation targets (I) the PWD and his/ her caregiver as well as (II) the treating GP. For the PWD the pharmacist analyses:

a) drug administration (support in drug administration - is there a caregiver or a nursing service involved?),

a) correct drug intake modality (time between drug administration and meal, time of day, frequency) and

a) an adherence check (what types of adherence problems are relevant for the PWD?)

For the GP the pharmacist checks (from a pharmaceutical point of view!!!):

a) correct drug choice and combination

a) potentially inappropriate medications

a) dosage

a) clinically relevant drug-drug interaction [38,39]

a) plausibility of reported adverse drug events and adherence

Step 2 Pharmaceutical recommendation

The pharmacist makes suggestions for the adaption of the pharmacotherapy: identified clinically relevant drug-drug interactions, appropriate drug choices, duplicate medications with a special focus on drugs from similar therapeutic classes, adequate pharmaceutical formulation, unclear or inconsistent application schemes etc. In cooperation between GP, DCM and pharmacist these recommendations are discussed.

Step 3 Application of recommendations

The GP is responsible for pharmacotherapy and initiates and monitors this. He is provided with all information about pharmaceutical interventions as well. Pharmaceutical interventions with the patient can comprise counseling of the patient and caregiver about correct drug usage, allocation of a drug dispenser, the provision of information leaflets about correct drug intake (short version of the summary of product characteristics), written adherence support strategies. If no caregiver is available, the nursing service monitors drug intake in the patient’s household by the GP. Documentation of all recommendations and the delivery of written information is mandatory and in this study we use an adapted version of a documentation sheet for home visit patients [32].

**Figure 1:**
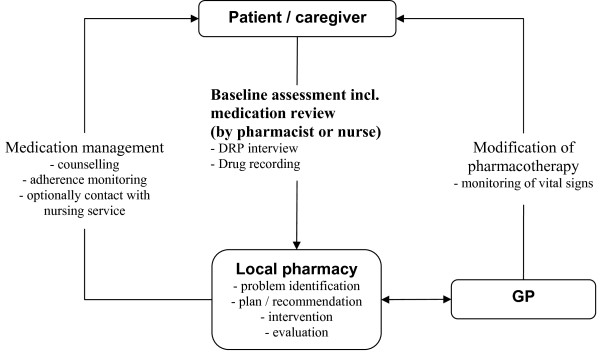
Recommendation for a workflow of the medication management for patients with dementia.

At a scheduled point in time, the home medication review is repeated to evaluate the success of the medication management process. An overall workflow of the medication management in the DelpHi study is provided in Figure 1. The recommendations for the content of general as well as dementia specific medication management are provided in Table 1.

**Table 1:** Recommendations for the content of general as well as dementia specific medication management

***Content of basic module***	***Content of disease specific module***
Medication review	Check for anticholinergic drugs
Activities for correct administration/storage	Check for falls increasing drugs
Regular (every 6 months) check by the local pharmacist	Monitoring of drug usage by a caregiver or nursing service, resp.
activities to improve adherence	Check for clinically relevant drug-drug interaction
Check for adverse drug events	Adherence-monitoring: pill counting by the pharmacy
Check for potentially inappropriate medication.	Supply of adherence supporting tools, as MEMS

### Quality management

The procedures of the medication management process are part of a standard operating procedure (SOP), which is updated regularly in the study center. The SOP is explained, trained and handed out to the pharmacist and the GP, and is rechecked and updated regularly by the study’s physician and pharmacist.

As a part of the study procedure we provide a guideline-based pharmacotherapy card to the pharmacist that is compiled by physicians and pharmacists in the study centre and is updated in the study centre. The card is written in a practical format and provides a reference for the pharmacist in the medication management. As a part of the intervention such card should be of practical use. The card contains:

a. reminder information about frequent comorbidities

b. information about approved antidementia drugs and their dosage, bioavailability, renal elimination, available pharmaceutical formulations, CYP-metabolism, drug-drug interaction, common adverse effects, and contraindications

c. general information about therapy for common psychiatric/ neurologic comorbidities (i.e. agitation, depression, delusion, sleep disorders)

d. general information about DRPs (substances/ intake modalities that cause them, alternative substances/ intake modalities)

### Outcome measures

The MM in Delphi as described in this article is designed to deliver information about needs in pharmacotherapy and to trigger activities to reduce these needs. The target variables are:

a) intake of antidementia drugs (regular intake of an inhibitor of an acetylcholine esterase or a NMDA-antagonist)

b) potentially inappropriate medication e.g. anticholinergic drugs

c) number of potentially clinically relevant drug-drug-interactions according to the ABDA database [40]

d) adherence

## Discussion

The DelpHi approach for medication review employs comprehensive instruments and procedures in the primary care setting, and this approach is able to evaluate the efficacy of medication management for persons with dementia. This approach may overcome limitations of previous research and may be transferable into routine care.

Studies on the efficacy of medication management for PWD have focused on nursing homes or other institutionalized settings, or they did not focus on inappropriate drugs [41-43]. In a recent systematic review of the effects of medication review the low quality of most studies was criticized [44]. The most important criticism was the lack of sufficient randomization and the lack of assessing important clinical outcomes, such as hospitalisations [44]. The MM described here is designed for the setting of primary care, is comprehensive and by being implemented in the complex intervention in the DelpHi study yields a high quality (randomisation, assessment of clinical outcomes and such).

However, there are some challenges in this trial as well:

1 Standardisation

This study is conducted partially in routine care, involves different professions and relies on cooperation and communication. We assume that adherence to the study protocol and intervention may be an issue. Monitoring of data quality simultaneously on different levels is required. However, we use a computer-assisted data collection and management system. This system systematically supports the documentation of the MM. It also supports early detection of incomplete and implausible data. We also included partial integration of computerized decision support which is known to have positive effects, for example, on the detection of DRP [45]. We also qualify the participating professions extensively; give continuous support and issue SOPs as well as the pharmacotherapy card as a reminder tool.

2 Validity of data

Over the course of the progression of dementia, patients will lose their ability to answer our study questions. Thus, several monitoring instruments for drug therapy in the DelpHi study are based on the caregiver and do not require information solicited in personal interviews. Including the caregiver, the patient’s drug data record at the local pharmacy and the access to the GP’s medical record will help to improve the validity of our medication and study data.

Our MM will improve pharmacotherapy in PWD living at home. As one main benefit of a home medication review, all drugs taken, including OTC-drugs are recorded. The issue of a comprehensive medication list helps to identify several drug related problems [46]. If the patient would only be asked about the taken drugs or if only drugs from the medication plan were accounted for in the medication check, there would be a considerable risk of underreporting. We assume that this causes inadequate pharmacotherapy that will be not overlooked in our HMR and MM. Specific targets are the avoidance of potentially inappropriate medication as well as anticholinergic drugs. OTC drugs, which often have anticholinergic properties are often taken by elderly people and they frequently cause clinically relevant drug-drug interactions. Hence, knowing about OTC-drugs we can identify drug-drug-interactions and try to avoid them. Our MM will identify problems related to drug administration that can be encountered by providing additional assistance with drug administration from, for example, a professional care giver.

Our MM systematically supports and improves cooperation between professions that might be relevant for pharmacotherapy in Germany. The GP is responsible for pharmacotherapy of the PWD and is supported by a DCM and a pharmacist. There is evidence, that a pharmacist may have an important role in optimizing geriatric pharmacotherapy [47]. In the DelpHi study we use an electronic health record documentation that comprises drug documentation. Based on electronic data management, all relevant information can be available to all health care professionals involved in a patient’s care. Written documents are made available to the partners, recommendations are written down and interventions like changes in pharmacotherapy are documented. This yields comprehensive information and transparency to all partners.

We expect that the HMR and MM developed for DelpHi is transferable into routine care once it has proven its efficacy. It is implemented into a study close to routine care, incorporates already all professions that deal with pharmacotherapy of PWD in routine care and shows a high degree of standardisation. It is very specific and detailed in its procedures, complies with the state of the art guidelines for dementia care and probably requires just a few adaptations to be efficient in routine care. The MM can help the PWD by improving his/ her pharmacotherapy, help the caregiver by reducing DRP that affect him/ her; help the pharmacist/ GP by providing information/ recommendation not available in current routine care. In general, we expect the MM to have positive effects on the individuals as well as on the health care system.

## Abbreviations

DCM: Dementia care manager; DDI: Drug-drug interaction; DRP: Drug related problem; GP: General practitioner; HMR: Home medication review; MM: Medication management; OTC: Over the counter; PIM: Potentially inadequate medication; PWD: Person/ patient with dementia; SOP: Standard operating procedure.

## Competing interests

All authors declare that they have no competing interests.

## Authors’ contributions

All authors contributed to the design and development of the study protocol in their area of their special expertise. TF drafted the manuscript and developed the medication management module. JRT coordinates the study and has substantially contributed to the overall design of the study. DW has contributed to the development of the medication management module and supervises the MM in the DelpHi-trial. GA is responsible for the economical aspects of the design. IK and ST influenced the medical intervention as well as the psychiatric assessment of the study. WH is the principal investigator of the study, has substantially contributed to the concept of the study. All authors have read and approved the final manuscript.

## Pre-publication history

The pre-publication history for this paper can be accessed here:

http://www.biomedcentral.com/1471-2318/13/121/prepub
